# Effect of Change in Spindle Structure on Proliferation Inhibition of Osteosarcoma Cells and Osteoblast under Simulated Microgravity during Incubation in Rotating Bioreactor

**DOI:** 10.1371/journal.pone.0076710

**Published:** 2013-10-07

**Authors:** Lijun Wei, Yan Diao, Jing Qi, Alexander Khokhlov, Hui Feng, Xing Yan, Yu Li

**Affiliations:** 1 School of Life Science and Technology, Harbin Institute of Technology, Harbin, China; 2 School of Biology, Moscow State University, Moscow, Russia; Georgia Health Sciences University, United States of America

## Abstract

In order to study the effect of microgravity on the proliferation of mammalian osteosarcoma cells and osteoblasts, the changes in cell proliferation, spindle structure, expression of MAD2 or BUB1, and effect of MAD2 or BUB1 on the inhibition of cell proliferation is investigated by keeping mammalian osteosarcoma cells and osteoblasts under simulated microgravity in a rotating wall vessel (2D-RWVS) bioreactor. Experimental results indicate that the effect of microgravity on proliferation inhibition, incidence of multipolar spindles, and expression of MAD2 or BUB1 increases with the extension of treatment time. And multipolar cells enter mitosis after MAD2 or BUB1 is knocked down, which leads to the decrease in DNA content, and decrease the accumulation of cells within multipolar spindles. It can therefore be concluded that simulated microgravity can alter the structure of spindle microtubules, and stimulate the formation of multipolar spindles together with multicentrosomes, which causes the overexpression of SAC proteins to block the abnormal cells in metaphase, thereby inhibiting cell proliferation. By clarifying the relationship between cell proliferation inhibition, spindle structure and SAC changes under simulated microgravity, the molecular mechanism and morphology basis of proliferation inhibition induced by microgravity is revealed, which will give experiment and theoretical evidence for the mechanism of space bone loss and some other space medicine problems.

## Introduction

The effect of microgravity on the proliferation of osteoblasts has attracted much attention from the research community in recent years because of its great importance for the mechanism of space bone loss and some other space medicine problems. Much work has been done on the effect of microgravity on proliferation of osteoblasts. For example, X.Y. Zhang et al. found in 2000 that by increasing the number of cells in G1 phase and reducing the number of cells in G2/M phase, simulated microgravity can obviously inhibit cell proliferation [1]. P. Kossmehl et al. reported in 2003 that weightlessness induced apoptosis in normal thyoid cells and papillary thyoidcarcinoma cells via extrinsic and intrinsic pathways [2]. X.G. Wang et al. noticed in 2013 that miR-214 targets *ATF4* to inhibit bone formation [3]. However, the relation between cell proliferation regulation and change in cell structure needs further study before a very good understanding of the mechanism of cell proliferation regulation can be achieved. It is learnt through previous work that simulated microgravity can also induce the rearrangement, or even depolymerization of cytoskeleton of human cells [4]–[6]. And the relation between the change in cytoskeleton and the cell proliferation is still an open question. Therefore, we tried to study the effect of simulated microgravity on the change in spindle structure and the proliferation inhabitation of mammalian osteosarcoma cells and osteoblasts by keeping them in a rotating wall vessel (2D-RWVS) bioreactor.

Osteoblasts are mechanosensitive, and they can sense small deformations through their attachment sites [7]. Recent studies confirmed that intact microtubules are needed for the load-induced proliferation and differentiation [8]. The change in cytoskeleton under microgravity may occur in a sounding rocket [9]–[10], during parabolic flight [11], in a spacecraft [12]–[13], or under simulated microgravity on ground [14]. Mouse oocyte maturation is impaired under simulated microgravity because microtubules and chromosomes can not form a complete spindle during oocyte meiotic maturation [15]. However, whether there is a change in the spindle structure in somatic cells under simulated microgravity, and the regulatory mechanism of that process have not been fully clarified yet so far.

Spindle is a special structure of microtubules associated with the segregation of chromosomes during mitosis. Microtubules and other proteins can assemble themselves into a structure consisting of cilia, flagella, matrix, centrosome, and spindle, etc. Centrosome and spindle can form a mitotic apparatus, and the integrity of a spindle determines the accuracy of chromosome segregation in space and time, which is related to the maintenance of chromosome stability [16]. A change in spindle structure takes place in 3T3 cells after they are put under simulated microgravity for 72 hours [17]. The defects in spindle structure can be detected by spindle assembly checkpoint (SAC). MAD2 and BUBl are the main components of a SAC signaling pathway. Their expressions will increase to cause a cell cycle arrest when the spindle structure becomes abnormal. The deletion of either *Mad2* or *Bub1* can cause severe chromosome missegregation to further increase the chromosomal instability [16], [18].

On the basis of these findings, we assumed that microtubules can feel the change in tension and alter the spindle structure, and any change in the spindle structure can cause a change in the SAC expression, which results in a cell cycle arrest and proliferation inhibition. This study provides support for the above hypothesis.

## Materials and Methods

This study is carried out in strict accordance with the recommendations in the Guide for the Care and Use of Laboratory Animals of Chinese society of experimental animal. The protocol is approved by the ethical committee of the Heilongjiang Medical and Laboratorial Animal Center. All surgeries are performed under sodium pentobarbital anesthesia, and all efforts are made to minimize suffering.

### Cultures of Osteosarcoma Cells and Osteoblasts

Only five cell lines are now used for the present study. Osteosarcoma cells are MG-63,U-2 OS and Saos-2, osteoblasts-like cells with high chromosome instability and osteoblasts are hFOB1.19 and rat calvaria primary osteoblast with less chromosome instability. All these cells are model cell lines commonly used for space bone loses and other space medicine problems. MG-63, U-2 OS, Saos-2 and hFOB1.19 are all purchased from ATCC. MG-63 cells are cultured in DMEM (Gibco-BRL) with 10% (v/v) fetal calf serum (FBS, Gibco-BRL). U-2 OS cells are cultured in RPMI-1640 (Gibco, BRL) with 20% (v/v) FBS (Gibco-BRL). Saos-2 cells are cultured in DMEM and Ham’s F12 (Gibco-BRL) with 20% (v/v) FBS (Gibco-BRL). All the media is supplemented with antibiotics (streptomycin 100 µg/ml; penicillinG 100 U/ml). All the cells are incubated at 37°C in a humidified atmosphere with 5% CO_2_. Human osteoblasts cell line hFOB1.19 was transfected with SV40 large T antigen and cultured in DMEM and Ham’s F12 (Gibco-BRL) prepared at a ratio of 1∶1 with 2.5 mM L-glutamine (without phenol red), 0.3 mg/ml G418, and 10% FBS (Gibco-BRL). The cells are incubated at 33.5°C∼34.0°C in a humidified atmosphere with 5% of CO_2_.

Animal care and handling are conducted in accordance with policies on the care and use of animals promulgated by the ethical committee of the Heilongjiang Medical and Laboratorial Animal Center. Calvarias of Sprague-Dawley rats at 3 days of age are cut off under sodium pentobarbital anesthesia. And primary osteoblasts derived from fetal Sprague-Dawley rat calvaria are cultured in DMEM (Gibco-BRL) with 10% (v/v) FBS (Gibco-BRL) [19]. The cells are verified as osteoblasts by their morphology, alkaline phosphatase (ALP) staining, and formation of calcium nodules [20]–[21]. The morphology of cells is analyzed by means of inverted microscopy, and it is found that the dissociated cells are spindle-shaped, triangular or polygonal, with the typical morphological characteristics of osteoblasts (Fig. S1A). More than 90% of the cells are ALP-positive [22] (Fig. S1B and Table S1) and von Kossa-positive [23].

### Cell Culture in Rotating Wall Vessel Bioreactor

Clinostat is wildly used for cell culturing, tissue engineering and space biology research. The gravity vector to cells in a clinostat is constantly changing all the time. When the gravity direction changing interval is shorter than the minimum response time (MRT) of a cell, the effect of gravity does not work, and instead it produces an effect similar to that under microgravity.

Rotating Wall Vessel (2D-RWVS) bioreactor is used to create different culture conditions for this study, and it was originally developed by China Astronaut Research and Training Center. This two-directional, multi-sample cell experimental device can be used to investigate the effect of simulated microgravity on the ground [24]. The cells are seeded over a cover slip (22×26×0.5 mm) and cultured on the slip for 24 hours. The slip is then transferred into the bioreactor of 0.04 m in diameter filled with growth medium. According to equipment manual and reference, the samples are rotated around the horizontal axis at 30 rpm [24]. The cells cannot respond to any change in the rotational direction at this speed. So this is considered to be the effect of simulated microgravity. The rotation around the vertical axis is considered to be the rotation control (dynamic control group). The cells cultured in the RWVS bioreactor without rotation are considered as the untreated control (static control group). In this study, the results of the rotation control and untreated control group showed no significant difference, and so, only the results of the simulated microgravity and untreated control are shown in this paper for simplification.

### Cell Proliferation Analysis

Cells are seeded over a blood cover sheet (22×26×0.5 mm) with an intensity of 1.2×10^4^ and cultured on the slip for 24 hours, and then transferred into the rotation bioreactor as detailed in *2.2* above. The numbers of living cells are counted in the following 1∼7 days to establish the cell growth curves. After rotation culturing for 24, 48, 72 and 96 hours, the cells are harvested after trypsin digestion and centrifugation at 114 *g*, and then collected for MI, cell cycle, DNA content and apoptosis analysis. Each and every experiment is repeated for three times, and the culture medium is changed every three days.

For MI analysis, the cells undergo the same process of chromosome observation. The total number of cells and the number of mitotic cells are counted, and the MI is calculated using the following formula:MI (%) = (the number of mitotic cells/the total number of cells)×100%.

For cell cycle detection and DNA content analysis, the cells are harvested and kept fixed with 70% (v/v) ethanol (Sinopharm chemical reagent Co. Ltd, China) for more than 24 hours. The cells are stained with Propidium Iodide (PI) solution. The PI solution contains 1% (v/v) NP40 (Sigma, USA), 100 mg/mL sodium citrate (Sinopharm chemical reagent Co. Ltd, China), 1 mg/mL RNase A (DNA and protease-free, Fermentas, USA) and 0.5 mg/mL PI (Sigma, USA). The cells are then analyzed with a BD FACSCalibur to establish the cell-cycle histograms. At least 2×10^4^ events are recorded for each sample. Cell-cycle histograms are analyzed using a ModFit Cell Cycle Analysis Software V2.0 (Verity, Topsham, ME) to determine the percentage of cells for each phase (G1, S, and G2/M). DNA contents were analyzed using a CELLQuest Analysis Software (Verity, Topsham, ME).

For cell apoptosis detection, the cells are harvested in the same process of cell cycle detection, and then treated using FITC Annexin V Apoptosis Detection Kit I (BD Pharmingen, USA). The cells are washed twice with cold PBS and then suspended in 1×Binding Buffer at a concentration of 1×10^6^ cell/ml. 5 µl of FITC Annexin V and 5 µl of PI is added into 100 µl of cell solution. The cells are gently vortexed and incubated for 15 minute at RT(25°C) in the dark. 400 µl of 1×Binding Buffer is added into each tube. The DNA content histograms are analyzed using a ModFit Cell Cycle Analysis Software V2.0 within 1 hour. The following controls are used to set up compensation and quadrants: unstained cells, cells stained with FITC Annexin V (no PI), and cells stained with PI (no FITC Annexin V).

### Spindle and Centrosome Structure Analysis

The cells are kept fixed at room temperature with 4% PFA (Sigma, USA) for 15 minutes, permeabilized in 1% (v/v) Triton X-100 (AMERSCO, USA) for 10 minutes, and blocked with 5% BSA (Sigma, USA) for 1 hour. For spindle observasion, cells are incubated overnight at 4°C with mouse anti-α-tubulin antibody (1∶1000, Sigma, USA) diluted with PBS, and then incubated at 37°C for 1.5 hour with FITC-conjugated goat anti-mouse IgG (1∶100, Zhongshan Biotechnology Co. Ltd., China) diluted with PBS. For centrosome observasion, cells are incubated overnight at 4°C with rabbit anti-Centrin-2 antibody (Santa, 1∶300) diluted with PBS, and then incubated at 37°C for 1.5 hour with TRITC-conjugated goat anti-rabbit IgG (1∶100, Zhongshan Biotechnology Co. Ltd., China) diluted with PBS. Between those steps, the cells are washed three times with PBS. The cells are stained with DAPI (Sigma, USA) to visualize nuclei (blue), mounted in 90% (v/v) glycerol and analyzed using a confocal microscope.

The total number of spindles and the number of multipolar spindles observed are counted, and the ratio of multipolar spindles is calculated using the following formula:

Ratio of multipolar spindles (%) = (number of multipolar spindles/total number of spindles observed)×100%.

### Analysis of MAD2 and BUB1 Expressions using Western Blot Method

The cells in the logarithmic stage of growth are treated with a rotating wall vessel bioreactor as mentioned above. The total protein of the treated or control cells is extracted with RIPA buffer (150 mM sodium chloride, 1% Triton X-100, 0.5% sodium deoxycholate, 0.1% sodium dodecyl sulphate, 50 mM Tris, pH 8.0). Aliquots of the protein are separated with 12% SDS-PAGE and transferred onto PVDF membranes. The membranes are blocked with PBS-T containing 5% skim milk, and then incubated overnight at 4°C with primary antibody (MAD2 or BUB1, purchased from Abcam), and then incubated at 37°C for 1 hour with HRP-conjugated secondary antibody. An ECL Western blotting analysis system (Amersham Biosciences) is used to detect the substrates, and GAPDH is used for internal control. The relative expression values of MAD2 or BUB1 are normalized to the amount of GAPDH.

### RNA Interference

p*Silencer* 4.1-CMV neo Kit (Ambion, USA) is used to transfect the mammalian cells under study. p*Silencer* 4.1-CMV neo vector containing short hairpin interfering RNA (shRNA) against MAD2 mRNA sequence or BUB1 mRNA sequence through the online siRNA design system of Ambion. shRNA sequences (see supplemental Table S2) is constructed using p*Silencer*™ 4.1-CMV siRNA Expression System. The p*Silencer* 4.1-CMV neo Negative Control plasmid supplied with the kit is a circular plasmid to encode a hairpin siRNA, and its sequence is not found in mouse, human, or rat genome databases (according to p*Silencer* 4.1-CMV neo Instruction Manual, Ambion, USA ). The resulting vectors are then stably transfected into U-2 OS cells using Lipofectamine 2000 Transfection Reagent (Invitrogen, USA) in accordance with the manufacturer’s manual, and then selected with 700 µg/ml G418.

U-2 OS cells are stably transfected with pmEGFP-alpha-tubulin expression constructed using Lipofectamine 2000 in accordance with the manufacturer’s instructions (Invitrogen Corporation). pmEGFP-alpha-tubulin is kindly provided by Dr. Jan Ellenberg, EMBL, Heidelberg, Germany.

### Statistical Analysis

Chi-square test (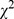-test) is used to test the differences in frequency distribution of incidence between the treated and control groups of multipolar spindle and MI. *T*-test is used to test the differences in number of cells and apoptotic cells between the treated and control groups. A *p*-value of less than 0.05 is considered as a statistical significance indicator. The data shown were obtained through three independent experiments.

## Results and Discussion

### Cell Proliferation Inhabition Under Simulated Microgravity

**Figure 1:**
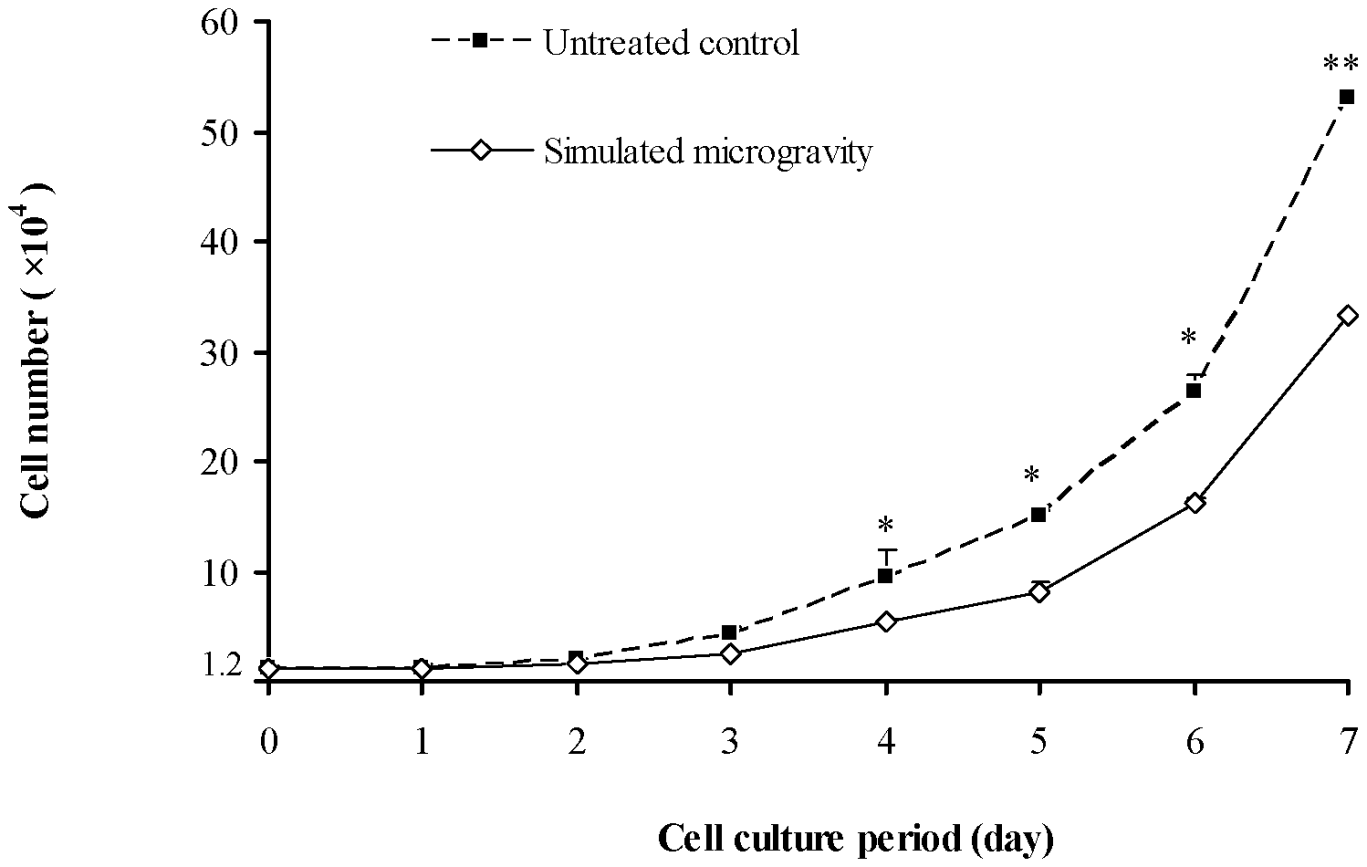
Cell growth curves of MG-63 under simulated microgravity. Numbers of living cells are counted in the following 1∼7 days. Cells grow into logarithmic phase before the 4th day on and simulated microgravity has its inhibition effect to cell proliferation with the extension of treatment time. After being treated for 4–7 days, the cell number of untreated control and simulated microgravity exhibit significant differences. The data shown represent the mean±S.E. (n = 3). *0.01<*P*<0.05, and **P<0.01 (t-test as compared to untreated control group).

In order to investigate the effect of simulated microgravity on the proliferation of osteosarcoma and osteoblast, cells are cultured under the gravity of 1 *g* and simulated microgravity to establish the cell growth curves. The inhibition of cell growth is observed under simulated microgravity for all the cell lines used for this research. As shown in Fig. 1, the cells grow into the logarithmic phase before the 4th day, and simulated microgravity has its inhibition effect on the proliferation of cells with the extension of treatment time. After being treated for four to seven days, the numbers of cells under untreated control and simulated microgravity exhibit significant differences (*t*-test, *P*<0.05).

It can be seen from these results that the cell growth of osteosaroma is inhibited, and the inhibition effect increases with the extension of treatment time, which is consistent with previous research results. According to the basic theory of cell biology, the inhibition of cell growth is mainly due to the decrease in cell formation or increase in cell death.

**Figure 2:**
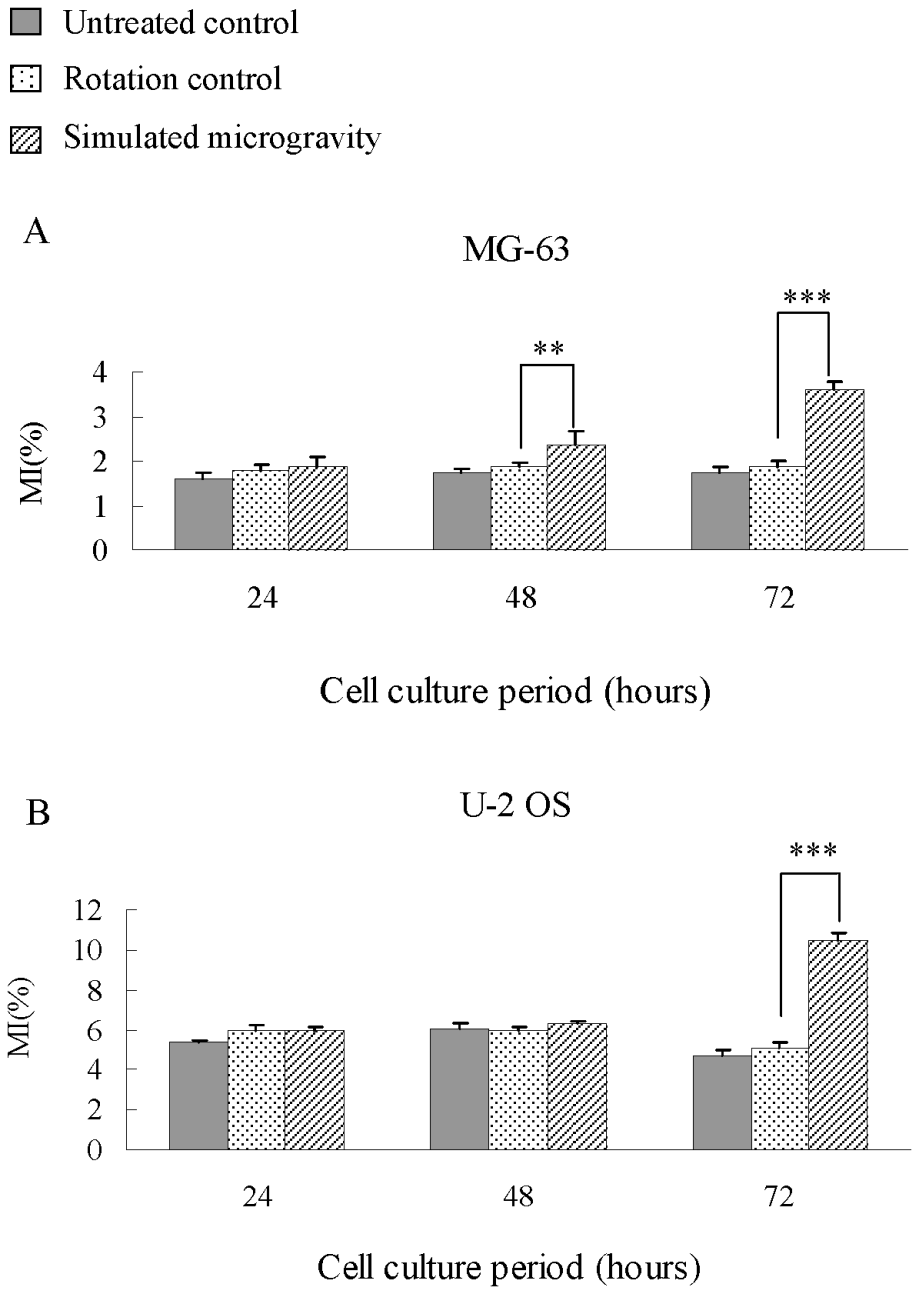
Mitotic index of MG-63 and U-2 OS cells under simulated microgravity. Significant increases in MI along with simulated microgravity duration, and significant difference resulting from being cultured for 48 and 72 hours in MG-63 (A) and 72 hours in U-2 OS (B). The data shown represent the mean±S.E. (n = 3). *0.01<*P*<0.05, **P<0.01, and ***P<0.001 (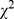-test as compared to rotation control group).

In order to see whether cellular reproduction level is affected by simulated microgravity, mitotic index (MI) is analyzed for this research. As shown in Fig. 2, the MI changes follow the same tendency for both MG-63 and U-2 OS cell lines. After being treated for 24, 48 or 72 hours, there is no significant difference (chi-square test, *P*>0.05) between untreated control and rotation control group. And after being treated for 24 hours, there is no significant difference between rotation control group and simulated microgravity group. While after being put under simulated microgravity for 48 hours, MI shows significant changes. And after being put under simulated microgravity for 72 hours, MI shows an extremely significant difference (chi-square test, *P*<0.001). It can be seen from these results that MI increase becomes more significant with the extension of treatment time.

The cause for the increase in MI can be interpreted using stimulated cell proliferation or accumulation of cells during mitotic (metaphase arrest). It can be seen from the cell growth curves that the increase in MI during the experiment can be contributed to the accumulation of cells in metaphase, which means a metaphase blockage.

### Cell Cycle Arrested and Cell Apoptosis Increased Under Simulated Microgravity

**Figure 3:**
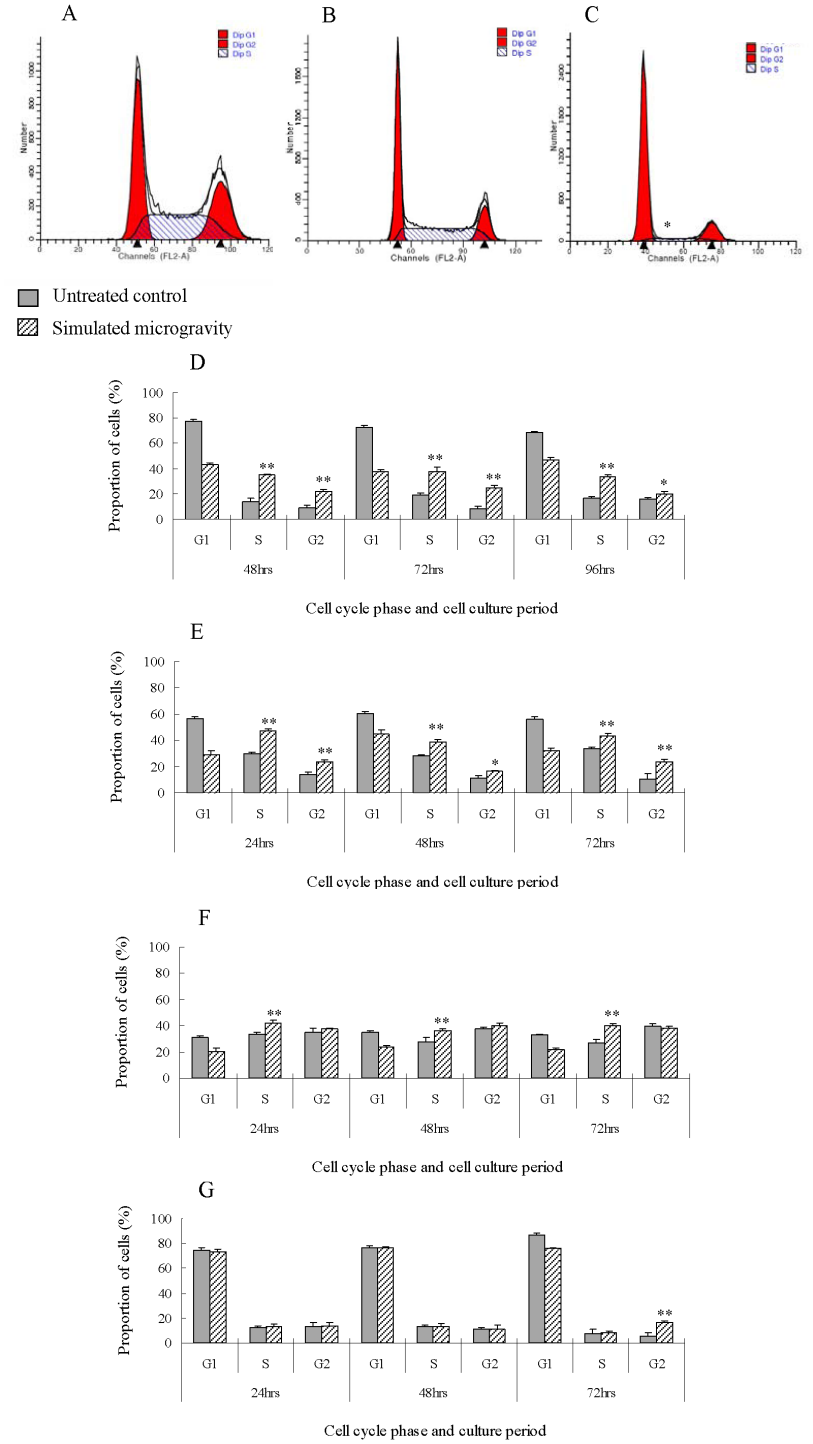
Results of cell cycle analysis. Cell-cycle histograms of MG-63(A), U-2 OS (B) cells and rat calvaria primary osteoblast(C) under simulated microgravity for 72 hours. The cycle changes of MG-63(D), U-2 OS(E), hFOB1.19(F) cells and rat calvaria primary osteoblast(G) under simulated microgravity. For MG-63 and U-2 OS the cell number in G1 phase decreases and increases in S and G2/M phase under simulated microgravity. For hFOB1.19 cells, increase in S phase under simulated microgravity. Rat calvaria primary osteoblasts increases only in G2 phases under simulated microgravity for 72 hours. The data shown represent the mean± S.E. (n = 3). *0.01<*P*<0.05, and **P<0.01 (*t*-test as compared to untreated control group).

In order to find out the reason why cell proliferation is inhibited, and verify the assumption based on MI, cell cycle and cell apoptosis are analyzed using FACSCalibur. The results of cell cycle analysis follow the same tendency in MG-63 and U-2 OS, which means, after being put under simulated microgravity for 24–96 hours, the number of cells in G1 phase decreases by more than 15%, and increases by more than 10% and 4% in S and G2/M phase, respectively (Fig. 3A–B). These results are inconsistent with those of Zhang (2000) [1] and Dai (2007) [25], so we repeated the experiments to prove the correctness of our results. It was found through further analysis that the cell lines used by Zhang and Dai’s are different from ours. So, we think the inconsistence is related to the cell lines used for the present study. The increase in number of cells in G2/M phase is consistent with MI, which is the right reason for MI increase. It can be seen from the results of MI and cell growth curves, that the cell cycles of MG-63 and U-2 OS are arrested in S and G2/M phases.

After being put under simulated microgravity, hFOB1.19 cells increases by more than 9% in S phase (Fig. 3C). After being put under simulated microgravity for 72 hours, rat calvaria primary osteoblasts increases by more than 10% only in G2/M phases (Fig. 3D). These results indicate that the cell cycle of hFOB1.19 is arrested in S phase, and after being put under simulated microgravity for 72 hours rat calvaria primary osteoblasts is arrested only in G2/M phases. This means simulated microgravity increase the cell numbers at different phases for different cells. For most of cells, arrest occurs in G2/M phase, which is consistent with the increase in MI that we observed. Experimental results indicate arrest can occur in other phase, for example, S phase, and cell cycle arrest lead to growth inhibition.

**Figure 4:**
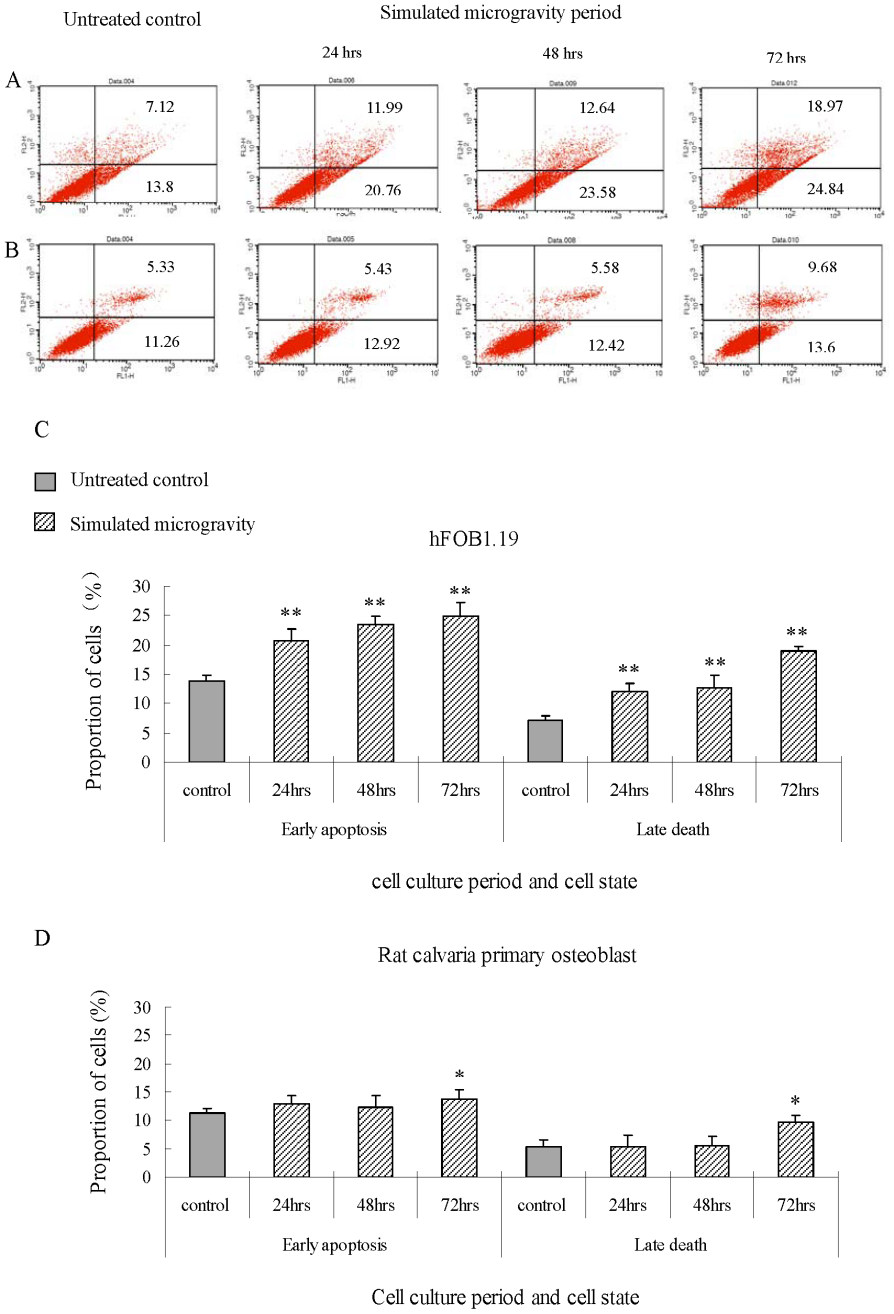
Apoptosis of hFOB1.19 cells and rat calvaria primary osteoblast under simulated microgravity. In hFOB1.19 cells (A and B), early apoptosis and late death mean extremely significant differences between untreated control and simulated microgravity for 24–72 hours. In rat calvaria primary osteoblasts (C and D), early apoptosis and late death mean significant differences between untreated control and simulated microgravity for 72 hours.The data shown represent the mean± S.E. (n = 3). *0.01<*P*<0.05, and **P<0.01 (*t*-test as compared to untreated control group).

Cell apoptosis is further analyzed for hFOB1.19 and rat calvaria primary osteoblast, as shown in Fig. 4. After being put under simulated microgravity for 24–72 hours, early apoptosis increases by more than 6% in hFOB1.19 cells. After being put under simulated microgravity for 24–72 hours, late death increases by more than 4%, which means there is an extremely significant difference (*t*-test, *P*<0.01) between untreated control and simulated microgravity. After being put under simulated microgravity for 24–72 hours, early apoptosis increases by less than 2% in rat calvaria primary osteoblast. After being put under simulated microgravity for 24–48 hours, late death does not change, but after being put under simulated microgravity for 72 hours, late death increases by more than 4% in rat calvaria primary osteoblasts, which means there is a significant difference (*t*-test, *P*<0.05) between untreated control and being put under simulated microgravity for 72 hours.

It can be seen from the results that simulated microgravity can cause an increase in extent and time of the apoptosis for different osteoblast. It can be seen from the cell cycle and apoptosis that the decrease in proliferation is due to cell cycle arrest and increase in apoptosis.

### Changes in Spindle Structure and Increase in the Incidence of Multipolar Spindles Under Simulated Microgravity

**Figure 5:**
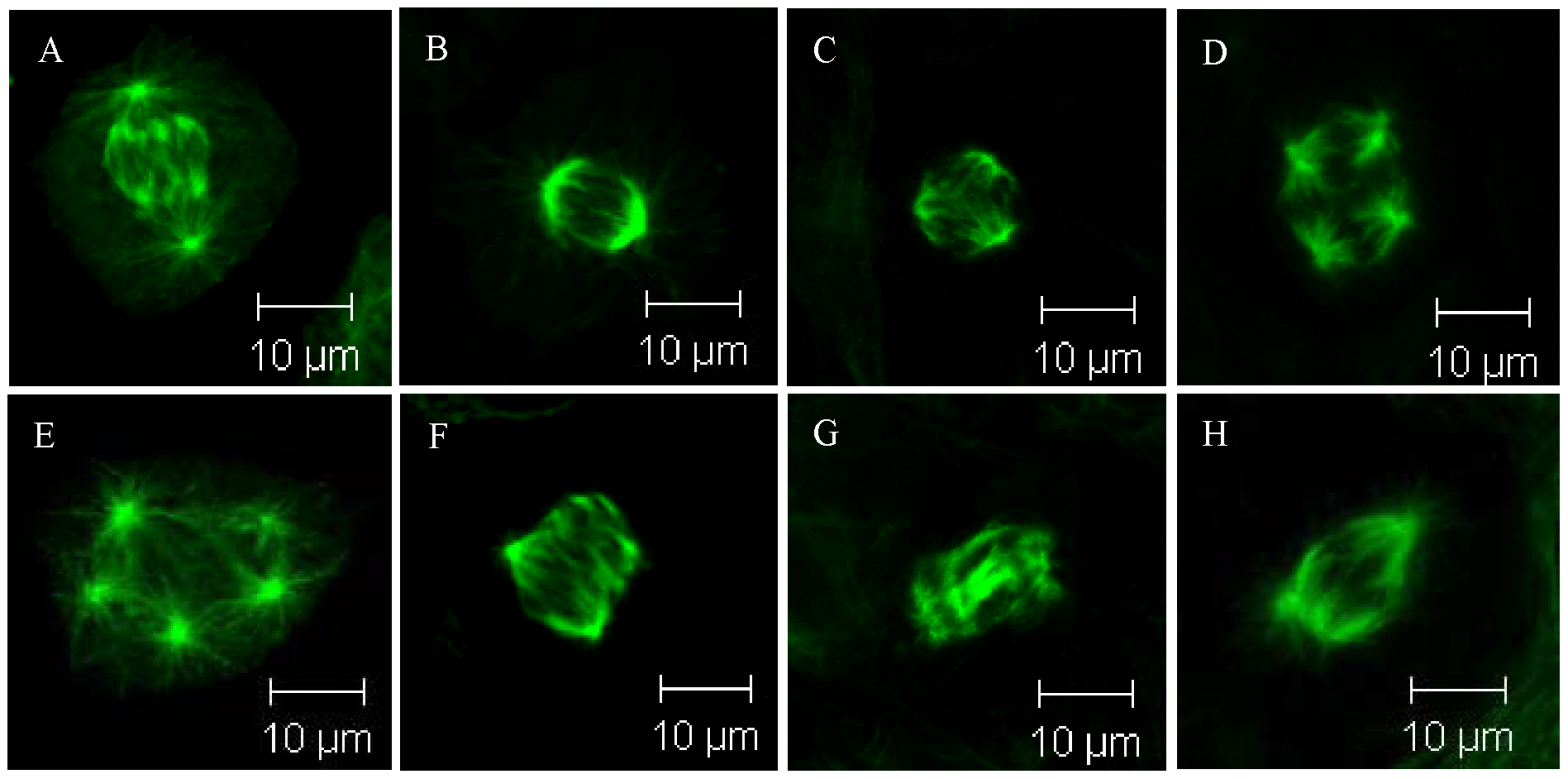
Spindle morphology in osteosarcoma and osteoblast cells. The spindles are stained green by anti-α-tubulin first antibody and FITC-conjugated goat anti-mouse IgG. A and B. Bipolar spindle of MG-63(A) cell and rat calvaria primary osteoblast (B) under 1 g condition. C–E. Multipolar spindle under simulated microgravity in MG-63 cell (C. three polar spindles, D. four polar spindles) and hFOB1.19 cell (E. five polar spindles). F–H. Microtubule arrangement change under simulated microgravity (F. MG-63, G. U-2 OS, H. rat calvaria primary osteoblast).

It is also observed through this study that cell proliferation inhibition is accompanied by cytoskeleton and spindle changes. We come to the conclusion which is similar to those of previous work that cytoskeleton changes under simulated microgravity. A spindle, which is the special formation of microtubules and the characteristic of mitosis, is observed under simulated microgravity during this study. It can be seen from these results that the spindle microtubules are distorted, the spindle changes in structure, and multipolar spindles appear (Fig. 5).

**Figure 6:**
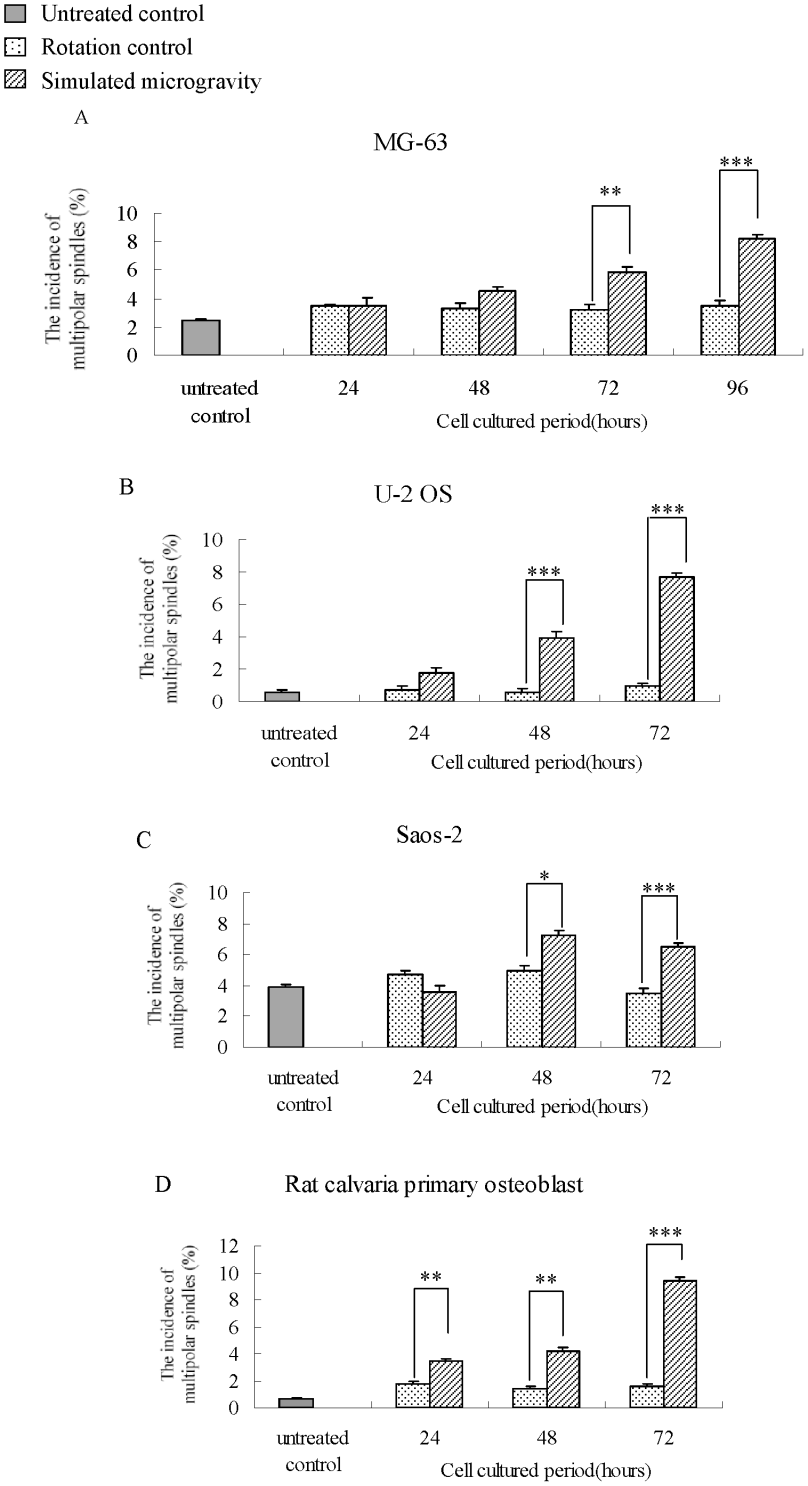
Incidence of multipolar spindles in MG-63, U-2 OS, Saos-2cells and in rat calvaria primary osteoblasts under simulated microgravity. The results demonstrate significant increase in multipolar spindle incidence along with simulated microgravity duration, and exhibit significant difference resulting from being cultured for 72 hours(MG-63, A), 48 hours(U-2 OS(B) and hFOB1.19(C)), or even 24 hours(rat calvaria primary osteoblast, D). The data shown represent the mean±S.E. (n = 3). *0.01<*P*<0.05, **P<0.01, and ***P<0.001 (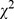-test as compared to rotation control group).

There are two polar regions for a normal spindle, but, in human osteosarcoma cells, multipolar regions may appear in one spindle. In order to find out the influence of simulated microgravity on the formation of a multipolar spindle, the ratio of multipolar spindles is calculated after the cells are cultured under simulated microgravity for 24, 48, 72 and 96 hours. As shown in Fig. 6, the incidence of multipolar spindles under simulated microgravity is higher than those for both untreated control and rotation control. Besides, the incidence of multipolar spindles increases with the extension of treatment time. Since there is no significant difference in the incidence of multipolar spindles between rotation control group and untreated control group, it can be concluded that the rotation around the vertical axis does not have any effect on the formation of a multipolar spindle.

It can be seen from the presentation above that the incidence of multipolar spindles is different for different human osteosarcoma cell lines, and the incidence of U-2 OS is the lowest. According to ATCC (http://www.atcc.org), the chromosome number of human osteoblast is not stable, but it is generally accepted by most of the researchers working in this field that hFOB1.19 is more stable than human osteosarcoma cell. However, as a matter of fact, multipolar spindles are observed in hFOB1.19 cells under normal culture conditions. In order to compare the responses of cell lines with different genome stability to simulated microgravity, primary osteoblasts are derived from Sprague-Dawley fetal rat calvaria. Although primary osteoblasts are more stable than other cell lines used for this study, multipolar spindles are also observed in the cells, which confirms that the availability of multipolar spindles is a common phenomenon in vitro. The incidence of multipolar spindles increases significantly after all the cell lines are put under simulated microgravity for this study.

**Figure 7:**
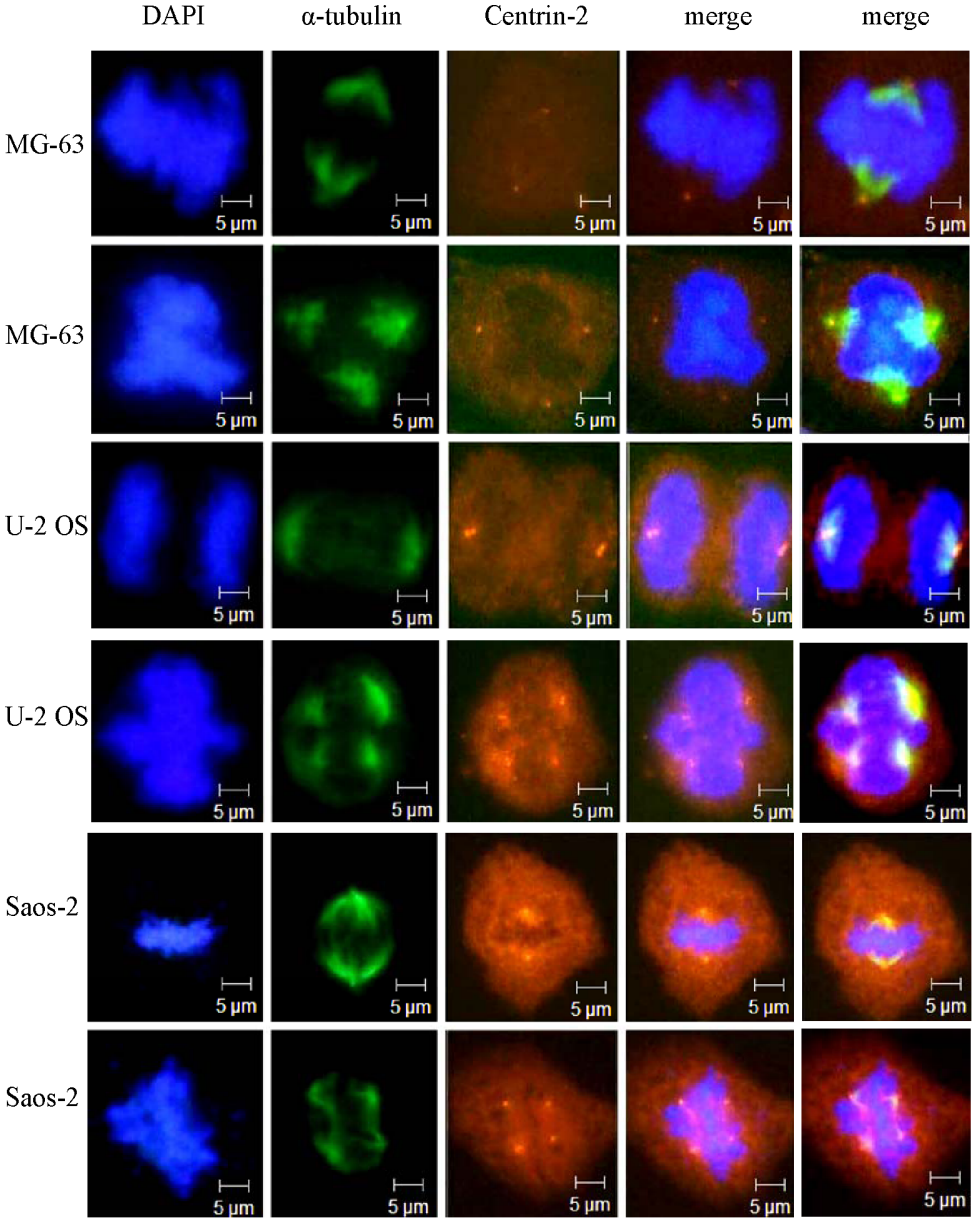
Centrosome status in human osteosarcoma cell lines MG-63, U-2 OS and Saos-2. MG-63 (line 1 and 2), U-2 OS (line 3 and 4), Saos-2 (line 5 and 6) cells are stained using immunofluorescence technique. Nuclei are shown in blue, microtubules in green and centrosome in red. The results indicate that multipolar spindles exist in both simulated microgravity and control groups, and centrosomes appear at each pole.

Normally, there is one centrosome in every spindle pole, but the spindle pole can be formed with the microtubules clustered without existing centrosome [26]. To further investigate the relationship between the formation of a multipolar spindle and the change in centrosome, U-2 OS, MG-63 and Saos-2 cells are analyzed with their centrosomes stained with anti-Centrin-2 antibody (Fig. 7). It is observed the centrosomes appear at each polar of a bipolar spindle and the multipolar spindle, supposing that a multipolar spindle appears simultaneously with multicentrosomes. There are centrosomes in all the polar regions indicate that simulated microgravity can change the number of spindle poles.

Spindle is an important apparatus for mitosis, and its change in structure can have its influence on the process of cell division, and therefore the cell proliferation. So we think that the abnormal structure of a spindle is related to the cell proliferation inhibition.

### Expression of MAD2 and BUB1 Increased Under Simulated Microgravity

SAC is a well-conserved mitosis control system, and it can be presented in kinetochore and monitors if the spindle structure and the chromosome display properly a bipolar attachment. If the attachment is not proper (monotely, syntely and merotely, etc.) [16], it triggers a cascade of reactions to inhibit the APC/C functioning by blocking the degradation of a target protein and the cleavage of a sister chromatid junction protein to stop the cell transition from metaphase to anaphase. This results in the arrest of cell cycle before the separation of a sister chromatid [18]. BUB1and MAD2 are two major proteins in SAC pathway, and they are located at the beginning and end of the pathway, respectively.

**Figure 8:**
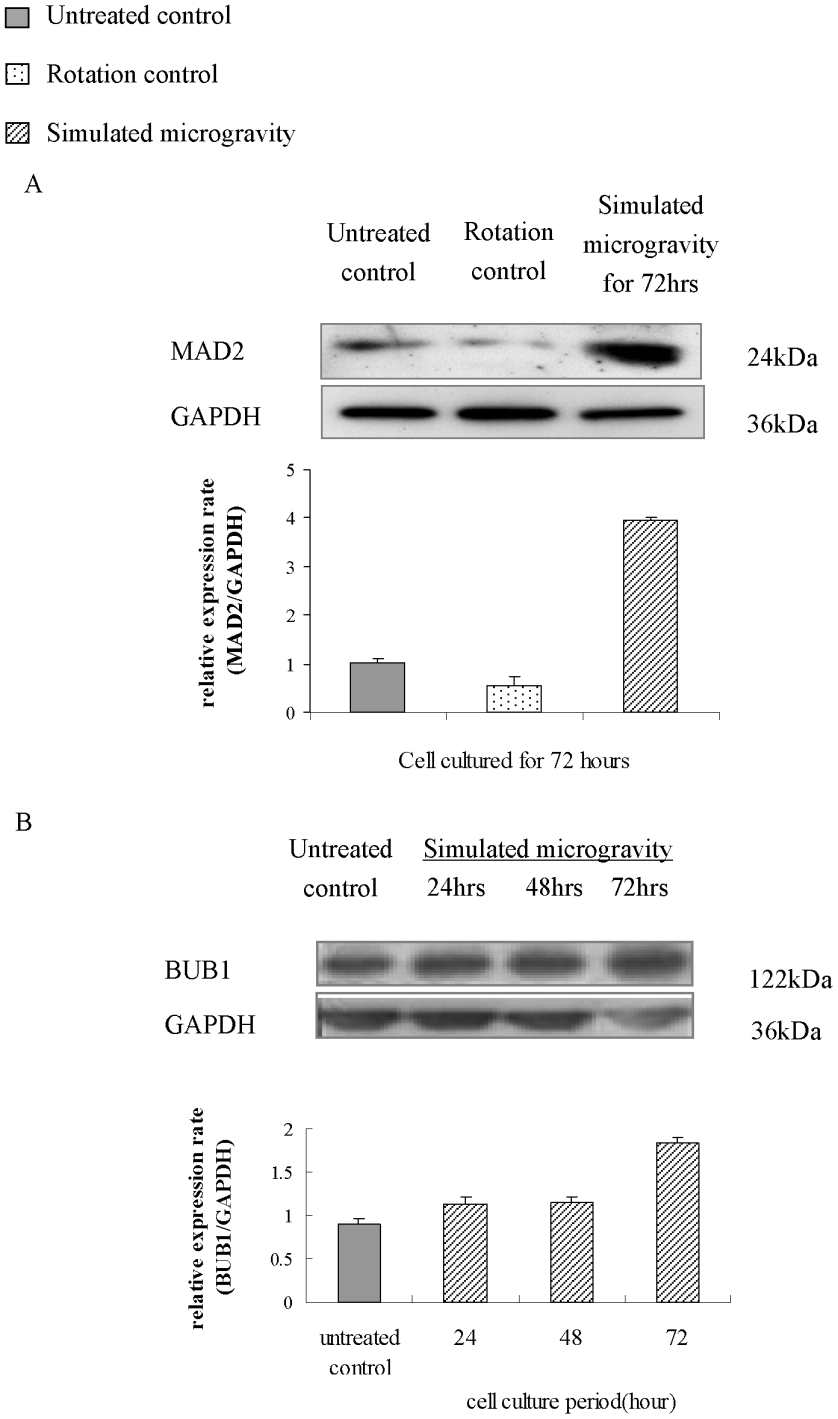
Results of Western Blot analysis of MAD2 in MG-63 cells after 72-hour cultivation and of BUB1 in MG-63 cells after 24, 48 and 72-hour cultivation under simulated microgravity. After MG-63 cells are treated with simulated microgravity for 72 hours, MAD2 and BUB1 expression increase to 3.9-fold (A) and 2-fold (B) as compared with untreated control. The relative expression rate shown represent the mean± S.E. (n = 3).

After the spindle pole changes under simulated microgravity, the expression of MAD2 and BUB1 are analyzed using western blot method in MG-63 and U-2 OS cells. It can be seen from the results that the expressions of MAD2 and BUB1 do not show a significant change in the rotation control group, but increase under simulated microgravity. After MG-63 cells were treated with simulated microgravity for 72 hours, the expressions of MAD2 and BUB1 increase to 3.9-fold (Fig. 8A) and 2-fold (Fig. 8B) in comparison with the untreated control, respectively. The expression of SAC protein increases with the incidence of a multipolar spindle. This induces the cell cycle arrest and enables the repair of cells. The erroneous attachments should be corrected during the cell cycle arrest. Anaphase will not occur until all the chromosomes form proper bidirectional attachments to spindle microtubules [16](Thompson et al., 2010). If the error cannot be corrected, the cells will be directed to apoptosis. If a SAC defect occurs, the cell will continue to divide, and it is very likely for a cell to undergo the malignant transformation and to lead to a high probability of genome instability [27].

On the basis of these findings, we assumed that simulated microgravity can induce a change in microtubule structure and a multicentrosome appears, and then lead to a change in spindle structure, SAC over expression, and cells blocked in the metaphase. The cells blocked in the metaphase have three fates, 1) divide after being repaired; 2) apoptosis because it cannot be repaired; 3) undergo abnormal division without being repaired. In 3.3 we have observed an increase in cell apoptosis, but we need further observation to confirm whether there is any other activity.

It is found through this study that, the expression of SAC proteins (MAD2 and BUB1) increase together with the changes of spindle structure, so that the abnormal cells are blocked in metaphase. That is why we detected more multipolar spindles in metaphase under simulated microgravity, which causes the inhibition of cell proliferation.

### The Cells with a Multipolar Spindle can Divide and Cell Division Increase in the Cells Knocked Down for MAD2 or BUB1

**Figure 9:**
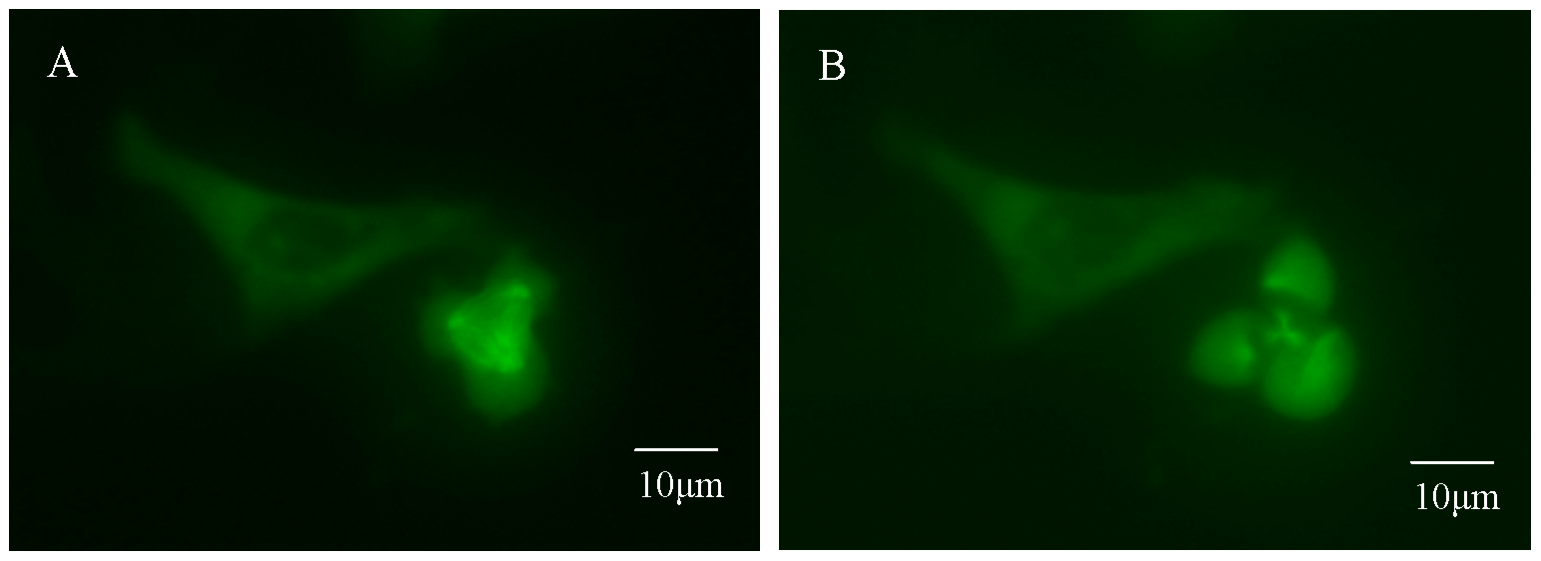
One U-2 OS cell with tripolar spindle divide into three parts. pmEGFP-alpha-tubulin is transfected into U-2 OS cells, and then living cells are observed by Workstation. The microtubules and spindles were found clear in green. Three-spindle cell (A) divides into three parts (B) in 10 minutes only.

In order to confirm the fate of multipolar spindle cells, pmEGFP-alpha-tubulin is transfected into U-2 OS cells, and then, living cells are observed through Workstation (Zeiss cell observer system on “Axio Observer Z1” microscope) for more than 24 hours. The microtubules and spindles in green are clearly observed. And some cells with a multipolar spindle can divide. For example, the three-spindle cell shown in Fig. 9 can divide into three parts in 10 minutes only. But most of the cells with multipolar spindle remain undivided for hours. It can be seen from these observsion that most of the cells with multipolar spindle are blocked in metaphase, and only a few of them can divide. Cell arrest is due to the abnormal spindle structure and the over-expression of MAD2 and BUB1. This is the reason why the incidence of multipolar spindle increases under simulated microgravity. The incidence of multipolar spindle is the result of dynamic changes between the formation and division of multipolar spindle cells. If more multipolar spindle cells are formed while less multipolar spindle cells divide, the multipolar spindle cells will accumulate in the culturing system. The number of cells will decrease as multipolar spindle cells divide.

In order to confirm the hypothesis mentioned above from the opposite, MAD2 and BUB1 are knocked down using RNAi technique, pSilencer™ 4.1-MAD2/BUB1 siRNA are transfected into U-2 OS cells as decribed in 2.6, and the incidence of a multipolar spindle and the change in cell cycle are detected in the cells knocked down for MAD2 or BUB1 under simulated microgravity.

**Figure 10:**
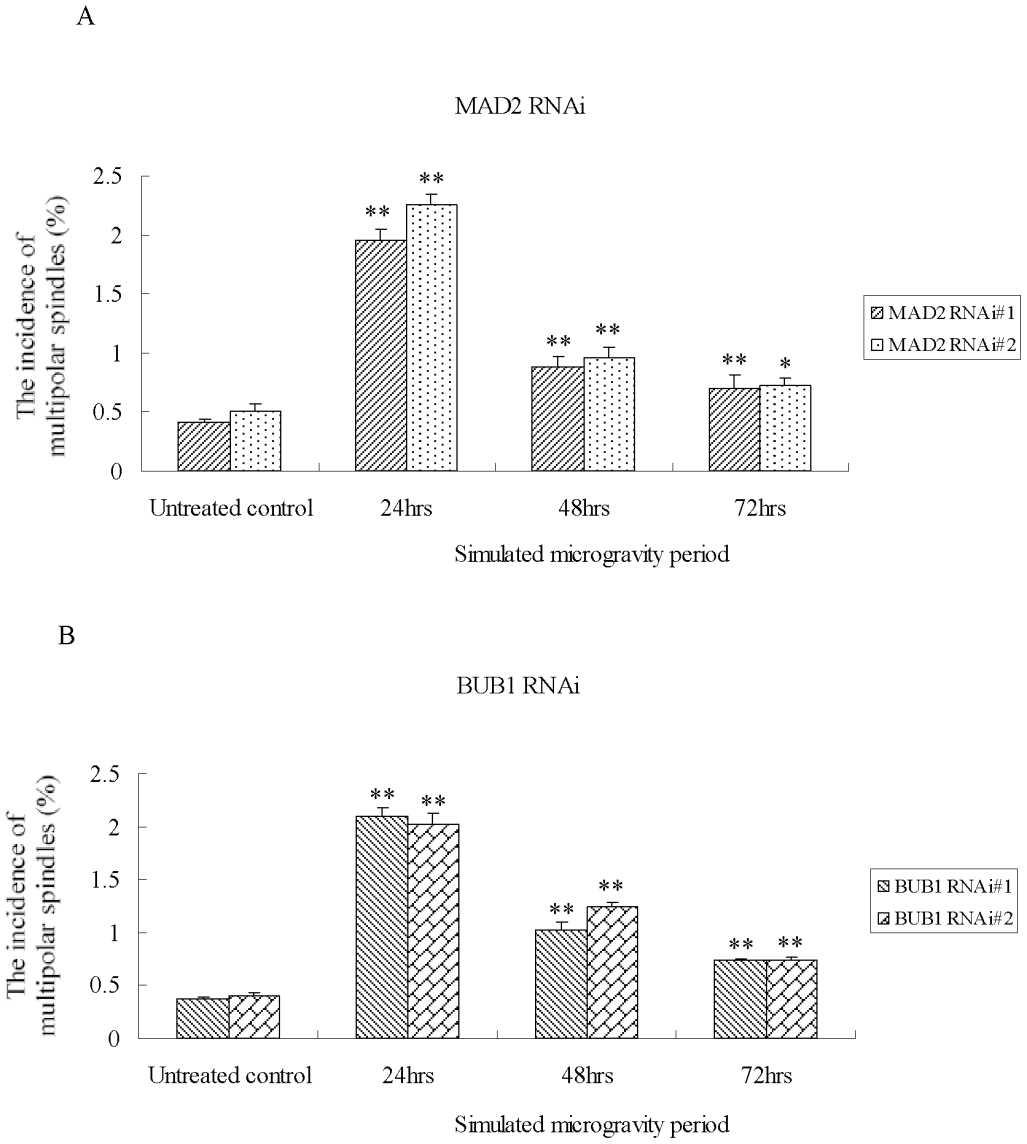
Incidence of multipolar spindles in U-2 OS cells after MAD2 and BUB1 (B knockdown under simulated microgravity. In MAD2 (A) or BUB1(B) knockdown cells, the incidences of multipolar spindles exhibit significant differences as compared with untreated control, but the increase level declines with time length for cell culturing. The data shown represent the mean±S.E. (n = 3). *0.01<*P*<0.05 and **P<0.01 (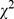-test as compared to untreated control group).

As shown in Figure S2, the changes in the incidence of a multipolar spindle in siRNA control group (sicon.) demonstrats the same pattern as the wild type U-2 OS, i.e., the incidence of multipolar spindles increases under simulated microgravity with the extension of treatment time. Among the cells knocked down for MAD2 or BUB1 (Fig. 10), under simulated microgravity, the incidence of a multipolar spindle exhibits a significant difference with respect to the untreated control, and the increase declines with the time of cultivation. After being put under simulated microgravity for 24 hours, the incidences of multipolar spindles in RNAi and sicon. groups reach the same high level. But after being put under simulated microgravity for 48 hours, the incidence of multipolar spindles of simulated microgravity group is much lower than that of sicon. group, but it is still higer than that of untreated control group. And after being put under simulated microgravity for 72 hours, the decrease in the incidence of multipolar spindles of simulated microgravity group will continue.

It can be seen from the results that multipolar cells divide after MAD2 and BUB1 knockdown, and lead to the entry of more cells into anaphase. DNA content is analyzed to see whether the cells can undergo multipolar division after MAD2/BUB1 knockdown. A normal division is completed when there is no change in the total DNA content, and the number of chromosomes remains unchanged in daughter cells. However the decrease in DNA content means one cell has divided into several cells.

**Figure 11:**
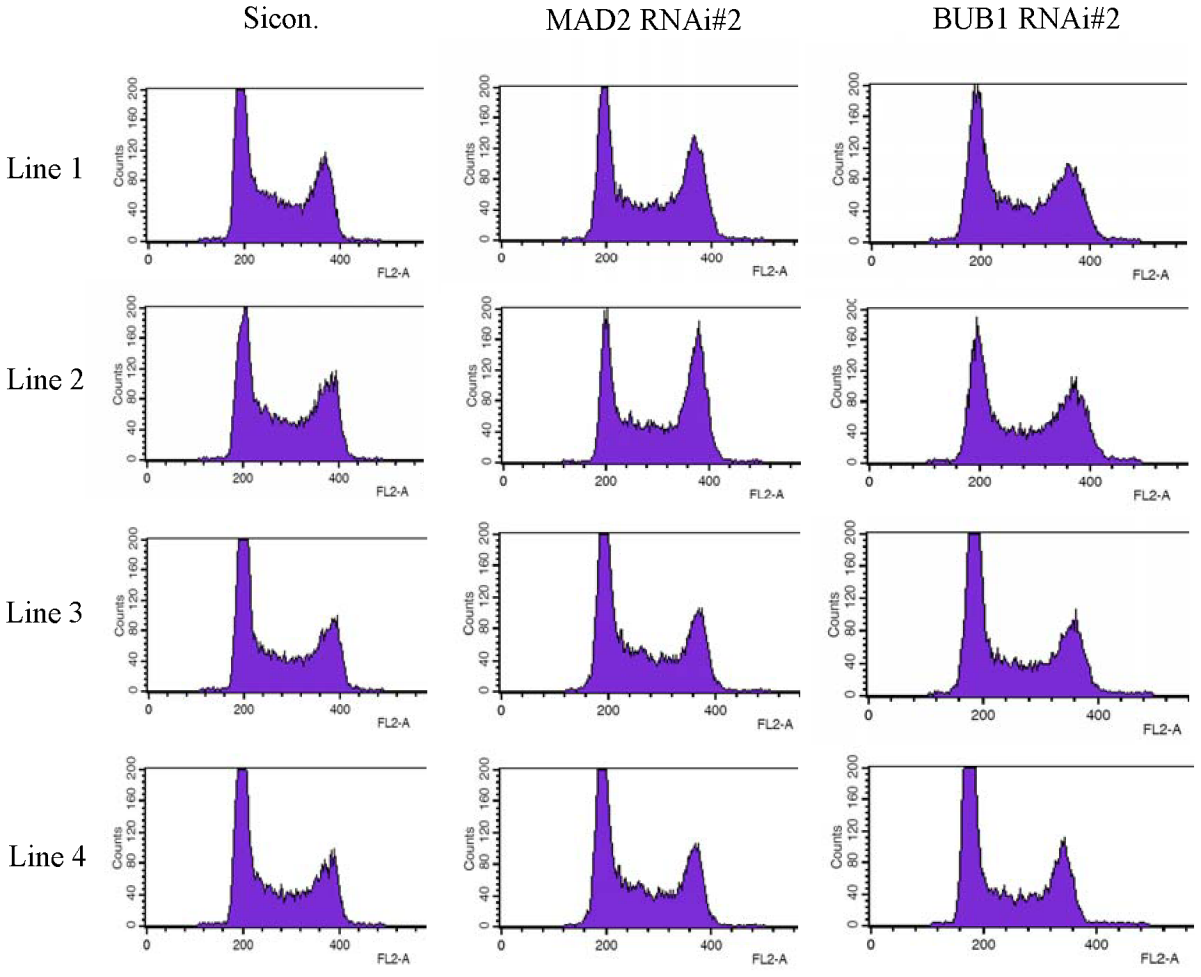
DNA contents in U-2 OS cells knocked down for MAD2 or BUB1 under simulated microgravity. Column 2. U-2 OS cells knocked down for MAD2. Column 3. U-2 OS cells knocked down for BUB1. Line 1. Untreated control (1 *g*). Line 2. Simulated microgravity for 24 hours. Line 3. Simulated microgravity for 48 hours. Line 4. Simulated microgravity for 72 hours. G1 peak stays at FL2-A 200 nm in sinco. group under different culture conditions, and is not time-varying. In the cells knocked down for MAD2 or BUB1, G1 peak also locates at FL2-A 200 nm under simulated microgravity for 24 hours, but shifts in the clone of MAD2 RNAi#2 and BUB1 RNAi#2.

DNA content is analyzed using FACSCalibur for U-2 OS cells knocked down for MAD2 or BUB1. As shown in Fig. 11 and Table 1, G1 peak stays at FL2-A 200 nm in sinco. group under different culture conditions, and it is not time-varying. In the cells knocked down for MAD2 or BUB1, G1 peak is also located at FL2-A 200 nm after being put under simulated microgravity for 24 hours, but G1 peak shifts to 188, 187, 176, 160 in the clone of MAD2 RNAi#1, MAD2 RNAi#2, BUB1 RNAi#1, BUB1 RNAi#2, respectively. It can be seen from the shift of G1 peak that the results indicated that the DNA content of diploid cells decreases, which confirms indirectly that multipolar division occurrs. Because the cell with a multipolar spindle divides into daughter cells, the decrease in DNA contents is due to the division of cells with multipolar spindle.

**Table 1:** FL2-A position of G1 peak under simulated microgravity (nm).

Treatment	Untreated control(1 *g*)	Simulated microgravity duration (hours)		
		24	48	72
sicon.	200	200	200	200
MAD2 RNAi#1	200	200	197	188
MAD2 RNAi#2	200	200	191	187
BUB1 RNAi#1	200	200	189	176
BUB1 RNAi#2	200	200	176	160

**Figure 12:**
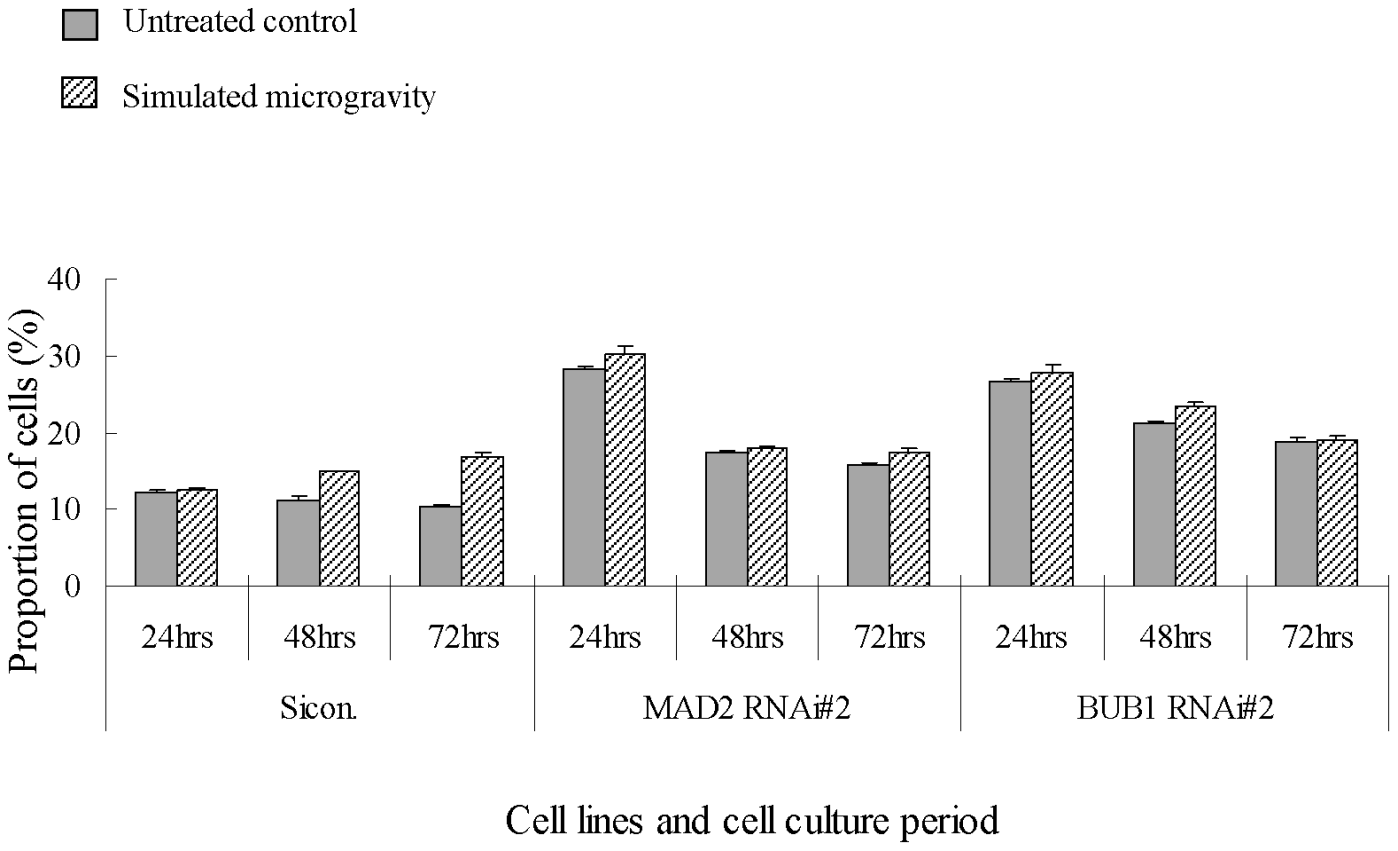
G2 phase changes in U-2 OS cells knocked down for MAD2 or BUB1 under simulated microgravity. For sicon. clone, the cell number in G2 phase increase with the extension of simulated microgravity, and 3.7% and 6.5% higher than untreated control under simulated microgravity for 48 hours and 72 hours, respectively. For MAD2 or BUB1 RNAi clones, the cell number in G2 phase under simulated microgravity is also higher than untreated control, but only 1–2% higher than untreated control.

Cell cycle is also analyzed using FACSCalibur in U-2 OS cells knocked down for MAD2 or BUB1. As shown in Fig. 12, in sico. group, the cell number in G2 phase increases with the extension of simulated microgravity, and 3.7% and 6.5% higher than untreated control under simulated microgravity for 48hours and 72hours, respectively. In MAD2 or BUB1 RNAi group, the cell number in G2 phase under simulated microgravity is also higher than that in untreated control, but only 1–2% higher than that in untreated control. These results follow the same change tendency of multipolar spindle cells, and also support the guess that multipolar spindle cells divide and the cell cycle arrest is relieved.

After the SAC proteins (MAD2 or BUB1) were knocked down, the number of multipolar spindles still increases under simulated microgravity, but, due to the defects of SAC, the cells can go on mitosis and lead to the decrease in DNA content, and cause the accumulation of cells with multipolar spindles was suppressed.

## Conclusions

In order to study the effect of microgravity on cell proliferation, mammalian osteosarcoma cells and osteoblasts were kept under simulated microgravity in a rotating wall vessel (2D-RWVS) bioreactor, and then the changes in cell proliferation, spindle structure, expression of MAD2 or BUB1, and effect of MAD2 or BUB1 on the inhibition of cell proliferation are investigated.

**Figure 13:**
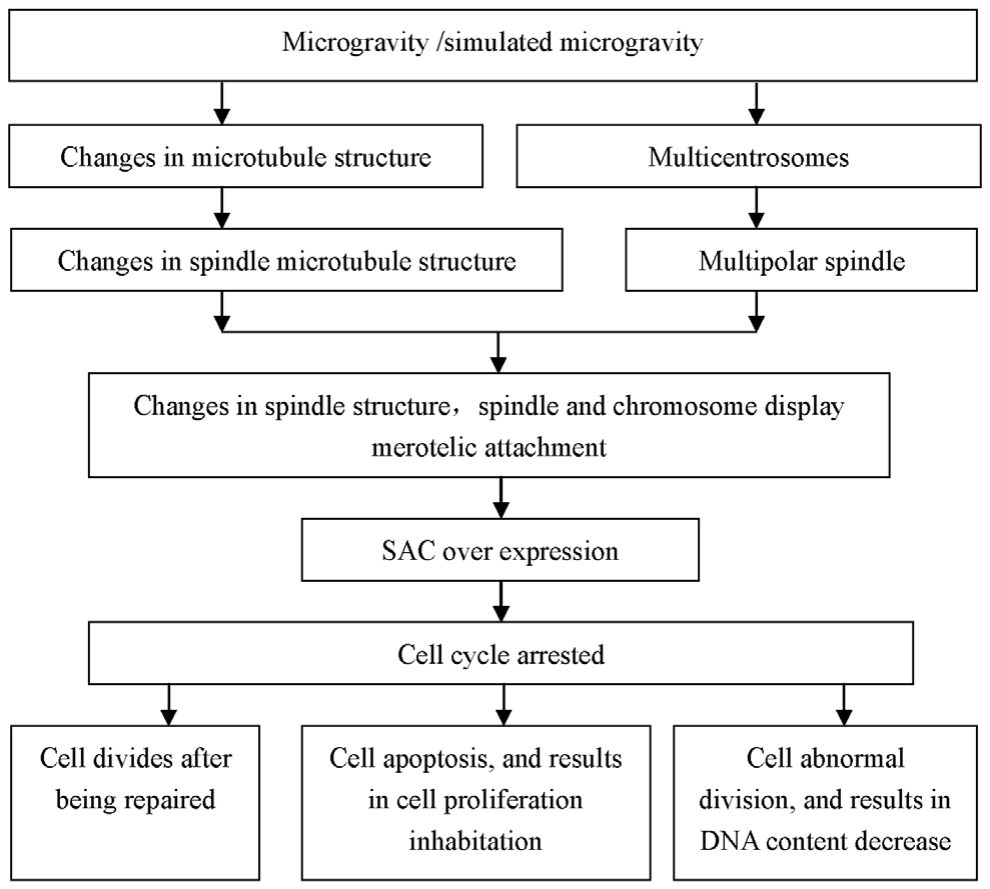
The possible pathway of cytoskeleton system “feels” the changes in tension and finally influences cell proliferation under simulated microgravity.

Experimental results indicate that the inhibition cell proliferation, incidence of multipolar spindles, multicentrosomes, and expression of MAD2 or BUB1 increases with the extension of treatment time. And multipolar cells enter mitosis after MAD2 or BUB1 is knocked down, which leads to the decrease in DNA content, and decrease the accumulation of cells within multipolar spindles. The process of cytoskeleton system “feels” the changes in tension and finally influences the cell proliferation under simulated microgravity by following the pathway below (Fig. 13). It can therefore be concluded that simulated microgravity can alter the structure of spindle microtubules, and stimulate the formation of multipolar spindles together with multicentrosomes, which causes the overexpression of SAC proteins to block the abnormal cells in metaphase, thereby inhibiting cell proliferation.

By clarifying the relationship between cell proliferation inhibition, spindle structure and SAC changes under simulated microgravity, the molecular mechanism and morphology basis of proliferation inhibition induced by microgravity is revealed, which will give experiment and theoretical evidence for the mechanism of space bone loss and some other space medicine problems.

## Supporting Information

Figure S1 Verification of osteoblasts by their morphology, ALP staining, and formation of calcium nodules. A. Rat calvaria primary osteoblasts before staining. The dissociated cells are spindle-shaped while some cells are triangular or polygonal, with typical morphological characteristics of osteoblasts. The nodule in the middle is one calcium nodule formed by the osteoblasts after cultivation for 15 days. B. Rat calvaria primary osteoblasts after ALP (alkaline phosphatase) staining. ALP produced by osteoblasts can react with cobalt nitrate and ammonium sulfide to form gray and black particles. The dark cells are osteoblasts, while the white ones are fibroblasts. C. Rat calvaria primary osteoblasts after calcium nodule formation detecting. The osteoblasts can produce the calcium nodules after cultivation for 15 days. The main chemical compound of the nodules is calcium phosphate. It can react with silver nitrate under UV light and produce black silver grains. Neutral red were used after silver nitrate staining and the osteoblasts are in red color.
(TIF)

Figure S2 Incidence of multipolar spindles of U-2 OS (sicon.) cells under simulated microgravity. The results are similar to those for wild type U-2 OS, that is, the incidence of multipolar spindles increases under simulated microgravity with time of cultivation.
(TIF)

Table S1 ALP staining analysis of the osteoblast culture.
(DOCX)

Table S2 shRNA sequences of MAD2 and BUB1 designed.
(DOCX)
