# Supplementary figures and images for: Effect of Change in Spindle Structure on Proliferation Inhibition of Osteosarcoma Cells and Osteoblast under Simulated Microgravity during Incubation in Rotating Bioreactor
Source: PLoS One. 2013 Oct 7;8(10):e76710. doi: 10.1371/journal.pone.0076710 (PMC3792057; doi:10.1371/journal.pone.0076710)

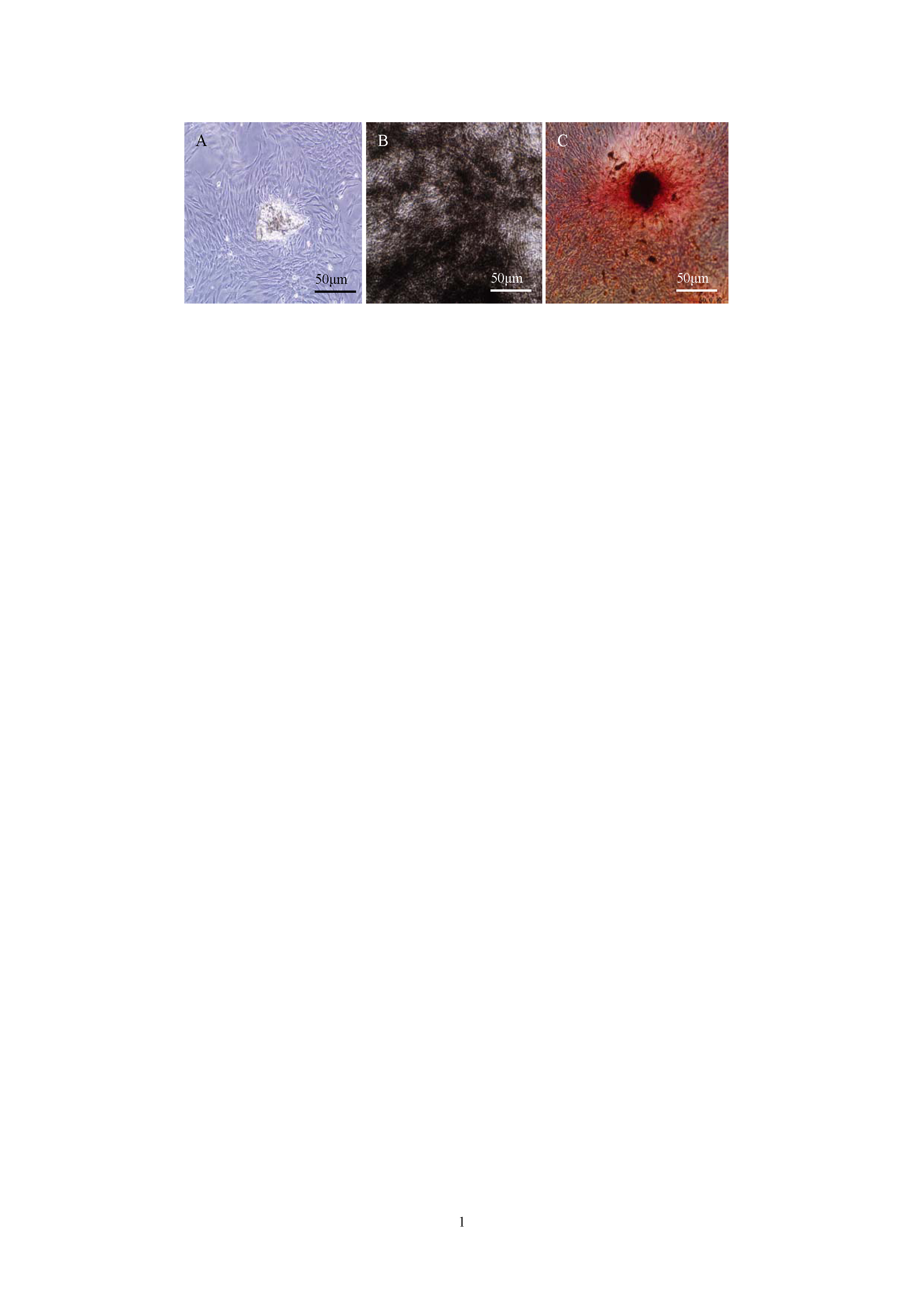

Supplement: Figure S1 — Verification of osteoblasts by their morphology, ALP staining, and formation of calcium nodules. A. Rat calvaria primary osteoblasts before staining. The dissociated cells are spindle-shaped while some cells are triangular or polygonal, with typical morphological characteristics of osteoblasts. The nodule in the middle is one calcium nodule formed by the osteoblasts after cultivation for 15 days. B. Rat calvaria primary osteoblasts after ALP (alkaline phosphatase) staining. ALP produced by osteoblasts can react with cobalt nitrate and ammonium sulfide to form gray and black particles. The dark cells are osteoblasts, while the white ones are fibroblasts. C. Rat calvaria primary osteoblasts after calcium nodule formation detecting. The osteoblasts can produce the calcium nodules after cultivation for 15 days. The main chemical compound of the nodules is calcium phosphate. It can react with silver nitrate under UV light and produce black silver grains. Neutral red were used after silver nitrate staining and the osteoblasts are in red color. (TIF) [file pone.0076710.s001.tif]

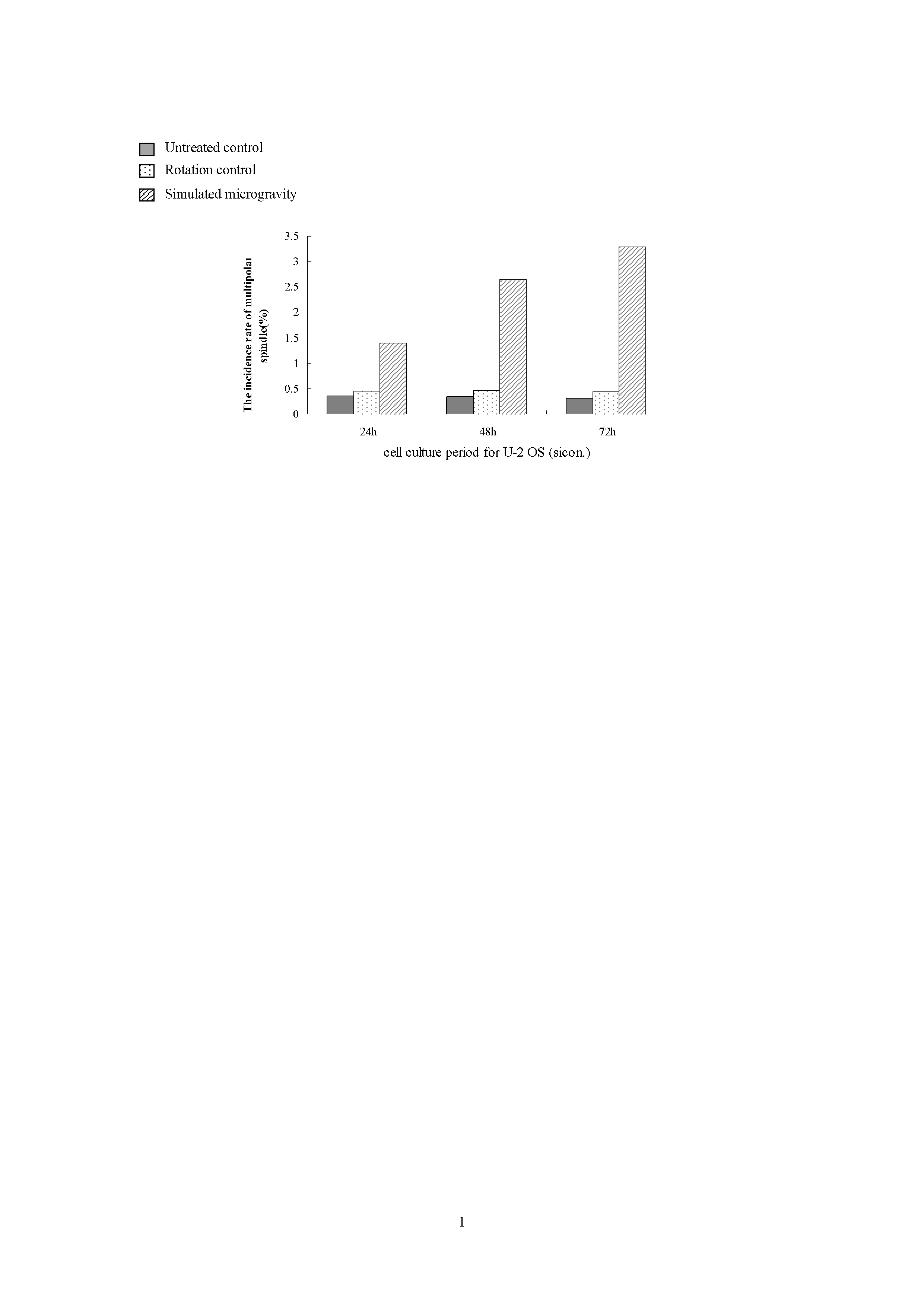

Supplement: Figure S2 — Incidence of multipolar spindles of U-2 OS (sicon.) cells under simulated microgravity. The results are similar to those for wild type U-2 OS, that is, the incidence of multipolar spindles increases under simulated microgravity with time of cultivation. (TIF) [file pone.0076710.s002.tif]
