# Supplementary material for: Effect of Change in Spindle Structure on Proliferation Inhibition of Osteosarcoma Cells and Osteoblast under Simulated Microgravity during Incubation in Rotating Bioreactor
Source: PLoS One. 2013 Oct 7;8(10):e76710. doi: 10.1371/journal.pone.0076710 (PMC3792057; doi:10.1371/journal.pone.0076710)
Supplement: Table S1 — ALP staining analysis of the osteoblast culture. (DOCX) [file pone.0076710.s003.docx]

Table S1 ALP staining analysis of the osteoblast culture

| Number of the microscope field of view (40×) | Number of ALP- positive cells | Total cell number | Osteoblast percentage* |
| --- | --- | --- | --- |
| 1 | 160 | 172 | 93.02% |
| 2 | 143 | 154 | 92.86% |
| 3 | 71 | 80 | 88.75% |
| 4 | 135 | 150 | 90% |
| Total | 509 | 556 | 91.55% |

*The experiment was repeated three times, and each time the results demonstrated ALP-positive cell percentage higher than 90%.
