# Supplementary material for: Effect of Change in Spindle Structure on Proliferation Inhibition of Osteosarcoma Cells and Osteoblast under Simulated Microgravity during Incubation in Rotating Bioreactor
Source: PLoS One. 2013 Oct 7;8(10):e76710. doi: 10.1371/journal.pone.0076710 (PMC3792057; doi:10.1371/journal.pone.0076710)
Supplement: Table S2 — shRNA sequences of MAD2 and BUB1 designed. (DOCX) [file pone.0076710.s004.docx]

Table S2 shRNA sequences of MAD2 and BUB1 designed

| shRNA | sequences |
| --- | --- |
| MAD2 RNAi#1 | 5'-GATCCGTGAAAGTGAGGAAATAAATTCAAGAGATTTATTTCCTCACTTTCACTTA-3'  5'-AGCTTAAGTGAAAGTGAGGAAATAAATCTCTTGAATTTATTTCCTCACTTTCACG-3' |
| MAD2 RNAi#2 | 5'-GATCCACAATGAAATATTGCTGTATTCAAGAGATACAGCAATATTTCATTGTTTA-3'  5'-AGCTTAAACAATGAAATATTGCTGTATCTCTTGAATACAGCAATATTTCATTGTG-3' |
| BUB1 RNAi#1 | 5'-GATCCCCAGGCTGAACCCAGAGAGTTCAAGAGACTCTCTGGGTTCAGCCTGGTTA-3'  5'-AGCTTAACCAGGCTGAACCCAGAGAGTCTCTTGAACTCTCTGGGTTCAGCCTGGG-3' |
| BUB1 RNAi#2 | 5'-GATCCGGTTGCCAACACAAGTTCTTTCAAGAGAAGAACTTGTGTTGGCAACCTTA-3'  5'-AGCTTAAGGTTGCCAACACAAGTTCTTCTCTTGAAAGAACTTGTGTTGGCAACCG-3' |
